# Remarks on Spinal Irritation, with Cases

**Published:** 1851-12

**Authors:** S. B. Hunt

**Affiliations:** Mendon, Monroe Co., N. Y.


### BUFFALO MEDICAL JOURNAL

AND

### MONTHLY REVIEW.

VOL. 7. DECEMBER, 1851. NO. 7.

### ORIGINAL COMMUNICATIONS.

ART. I.—*Remarks on Spinal Irritation, with Cases.* By S. B. Hunt, M. D., of Mendon, Monroe Co., N. Y.

Dr. Isaac Parrish, of Philadelphia, said, some twenty years ago, that, "by "a disorder in the normal actions of some portion, or the whole, of the spinal "marrow and sympathetic ganglia, either occurring primarily, or induced by "some pre-existing irritation; symptoms are manifested in different tissues, "producing diseases distinguished by numerous appellations, though usually "described by nosologists under the term *neuroses;* and, further, that all "these complaints are reducible to the two terms of Neuralgia and Hysteria."*

If this position can be supported, and if we can reduce to one pathology this lengthy list of functional disorders of the brain and nervous system, we are doing much to simplify the most complex portion of our nosology, and rendering its treatment comparatively simple and easy. Dr. Parrish, in the article above referred to, considers spinal irritation as the most frequent cause of neuralgia and hysteria. He brings to the support of this hypothesis a

* American Journal of Medical Sciences, for 1832.

number of previous writers, and deduces from their observations the fact that "tenderness on pressure over some portion of the spinal column, is an attend-"ant and prominent symptom in most chronic nervous complaints, and that "a removal of this tenderness by appropriate external applications, is the "most important indication in the treatment of the accompaning nervous "symptoms."

These quotations from Dr. Parrish are intended as a text upon which I may amplify, and to the support of which I shall bring a number of cases. It is, then, with the object of reaffirming views long since advanced, and of harmonizing the various doctrines and methods of treatment advanced by Drs. Player, Brown, Darwall, Teale, Tate, Parrish, and Chapman, that I offer this article. It will be seen that these writers have not attempted to explain in what consists the "disorder in the normal actions of the nervous system," spoken of — they have merely fixed its locale — they have shown that spinal irritation alone may produce the protean symptoms of neuralgia and hysteria, of epilepsy, catalepsy, and tetanus, of the whole host of disorders whose names end in "*algia*," "*atica*," and "*dynia;*"— they have also shown that irritation of the sympathetic ganglia may produce the same chain of diseases, — and they have proved angina pectoris to be a neuralgia of the pneumo-gastric nerve, but they have not told us in what this irritation consists. Here is a broken link, and he who can tell us what is "irritation," unites the chain, and brings this whole vexed question of nervous diseases into the region of philosophical induction and rational treatment.

I shall attempt to prove that spinal or ganglionic irritation is in many cases dependent upon circulatory derangement, either *congestive* or *inflammatory*. I do not suppose that it is necessary to prove that inflammation, or congestion, exists in *all* those parts most evidently affected in the course of nervous disease. It is a well understood fact, that diseases of the nervous centres manifest themselves by symptoms occurring at the extremities of the nervous trunks originating therefrom. The involvement of a nerve in the foot, in the cicatrix of a punctured wound, may produce all the frightful phenomena of tetanus, yet a post-mortem examination may reveal no evidence of inflammation, save in the cicatrix itself.

It seems to have been a settled point with writers, that *pain, without fever*, is the distinguishing characteristic of neuralgia. But it is doubtful whether every case of neuralgia, is not accompanied by more or less febrile action. Close inquiry will reveal the fact that a slight chill precedes the access of pain in periodic neuralgia. *All* cases of spinal irritation, so far as my observation extends, are subject to slight, but well marked chills generally occurring in the morning, and followed by heat of the skin, slightly accelerated pulse, rushes of blood to the head, coldness of the extremities, &c. Again, the treatment most successful is found to be more or less antiphlogistic in its character. M. Piorry urges that an inflammation may exist in the neurilemma or cellular tissue of a nerve, invisible to the eye in an autopsy, yet extensive enough to cause extreme suffering, and he supports his view by many cases where general, and more especially local bloodletting were alone sufficient for the cure of severe neuralgia. Those cases depending on abdominal irritation, and cured by the cathartic pill of Sir Charles Bell, are certainly instances of inflammatory action, manifesting itself upon a distant part by reflex irritation. No one will dispute the inflammatory nature of that class of cases called nervous rheumatism, and cured by calomel and opium, guiacum, and colchicum. Another instance is those cases of facial neuralgia cured by sal ammoniac and iodide of potassium, and depending upon periostitis around some decayed tooth. The revulsive treatment by blistering, cupping, and antimonial sores, is certainly antiphlogistic — lastly the use of quinine. Undoubtedly it acts directly in the relief of pain as a hypnotic, but I consider its modus operandi in neuralgia, to be identical with its action in cerebro spinal meningitis, in the congestive fevers of the west, and in remittent and intermittent fevers. It removes local plethora and congestions, and equalizes the circulation.

In the cases which I append, it will be noticed that I give a wide significancy to the term neuralgia. They are cases which fairly illustrate the two propositions which I have endeavored to make clear, viz:

1st. "That tenderness, on pressure, in some portion of the spinal column, is an attendant on many chronic neuralgic affections, and that by relieving it in the manner proposed, these complaints are either entirely eradicated, or temporarily suspended."

2d. That congestion, or imflammation, constitute the pathology of neuralgia.

Case 1st. J. S., a stout laboring man, aged fifty, had been for several days suffering with acute pain in the right side, accompanied by very high fever. His physician had been unable to make a diagnosis, but is inclined to consider it typhus fever. Pulse full, hard, and frequent, skin hot, tongue clouded. I found one of the dorsal vertebræ very tender. Gave him five grains quinine, and waited to watch the effect. In an hour he was in a state of profuse perspiration, and free from pain. Applied a blister to the spine. During the night pain and fever returned, and was again relieved by quinine. Dysentery followed in a few days, and there was evidence of great portal congestion. After his recovery he suffered from neuralgic pain in the calf of the right leg — (November, 1850.)

Case 2d. Mrs. C., a very nervous fidgetty woman, aged forty, recently confined. Found her in great distress with pain in the back of the head. Cords of the neck were tender and stiff. Pulse sharp and frequent, skin hot. Gave quinine, five grs., with sulph. morph. gr. one-eighth. This relieved her in a very short time, and the febrile action subsided with the pain —(March, 1850.)

Case 3d. April, 1851 — D. B., aged thirty-five, stout laboring man, has sciatica, with tenderness in the sacral region. Is easy during the night, but at 4, A. M., had a well marked chill, followed by slight fever, and intense pain in the sciatic nerve, running sometimes to the calf of the leg, and heel. Quinine given before the morning chill broke up this train of symptoms, but a return to labor reproduced them. I blistered the sacrum, gave cathartics, and renewed the quinine with benefit, but not gaining as fast as he wished, he went to a German physician in Rochester, who applied the potential cautery so thoroughly, that a large slough was produced. He came home in a fortnight cured.

Cases like the above are of very frequent occurrence, and I could multiply them *ad infinitum*, but they are sufficient as a type of simple congestive neuralgia, dependent on spinal irritation.

Case 4. 12th February, 1847, called to Mrs. O., aged thirty-five. Symptoms are those of "bilious colic." The usual remedies relieved her, but in the afternoon of the 13th, the pain returned and soon became alarmingly violent. Opiates and cathartics were given with a desperate energy, and in enormous doses, under the oft-repeated admonition of my consulting physician to "*bore her through somehow*." I bolted from the treatment on the fifth day, and Doctor Wells, of Castile, Wyoming Co., was added to the consultation. Doctor Wells advised milder means — thought the cathartics mischievous. He suspected peritoneal inflammation, but admitted the possibility of tonic spasm being the source of the constipation. A soothing treatment was agreed upon as covering both views of the case, and with a view to euthanasia. At this time we had the most horribly fœtid stercoraceous vomiting recurring every few hours. My note book describes her appearance at the fifth day, as follows: "She lays, with intervals of ease and rest, dozing half an hour at a time, pulse small and hurried, thick brown crust on the tongue, very slight tenderness of the bowels, and that confined to the descending colon, countenance hippocratic, and hiccoughing most of the time — mind tolerably clear. The colic pain now comes on in paroxysms between her naps, and lasting from ten minutes to an hour, and of fearful violence. The colon rises up like a huge snake, she screaming with agony." For several days there was no change, but she gradually improved. Her tongue cleaned, countenance and pulse improved, appetite returned, and stercoraceous vomiting occurred only twice *per diem.* Still no dejection from the bowels occurred till the fifteenth day of her illness, when the bowels moved freely, and she rapidly recovered. This lady had suffered much from an old spinal irritation, and I now think that it was a case of tonic spasm of the lower bowel, dependent upon spinal irritation; if so, counter-irritation to the spine, and a thirty gr. dose of quinine, would have been the best possible treatment. As it was, the treatment pursued for the last ten days was a judicious one. That for the first five days was a curious melange of colocynth, gamboge, scammony, calomel, hot baths, tobacco injections, mustard poultices, and opium. She lived through it, and the case is worthy of being preserved with a case of obstinate obstipation of the bowels, published in this Journal a few months since by the editor, which also seemed to be connected with spinal irritation.

Case 5. March 18th, 1851, called to Mrs. A. M., a lady aged about thirty years. She had suffered years before with a spinal disease, which left a lateral curvature at the fifth dorsal vertebræ. Her health for four years past, has been good. She has now neuralgia of the three branches of the fifth pair of nerves on the left side. The spine, in the intrascapular region, is quite tender to the touch. There is an excited state of the circulation which hardly merits the name of fever. ℞ quinine gr. 5, sulph. morph. gr. quarter. Advised a blister to the spine, but she would not consent to it. The paroxysm of pain became periodic, returning every morning at 11, A. M., unless anticipated by the above prescription, until the 17th, when at the usual hour of attack she was taken with cramp in the shoulders, with extreme pain. This assumed the character of tetanic spasm, with opisthotonos of the upper part of trunk and head. There were cramps in the hands and feet. There was no derangement of the menstrual function. Extremities cold, pulse regular. ℞ quinine 5 grs. every twenty minutes. Cupped the spine — frictions to extremities with mustard pediluvium. A blister was applied between the shoulders, and brandy sling, camphor, and carb. ammon, given as freely as the emergency seemed to call for. After an hour the spasm yielded and I left her cheerful and comfortable. At 3½, P. M., I found her again in tonic spasm, with trismus and general rigidity and opisthotonos. Mind clear. Repeated the treatment of the morning, adding sulph. morph. half gr., with ether and valerian, and a tobacco poultice to the epigastrium. There was a marked congestive action apparent. The rigidity lasted two hours without any cessation of convulsive action, being purely tetanic in its character. Profuse perspiration, with returning warmth, followed. Vomiting of greenish fluid commenced and kept up through the night. At 8, P. M., gave an enema and procured an evacuation from the bowels. There was great depression and fainting, and during the night I gave brandy, camphor, and carb. ammon, *pro re nata.* During the 18th only some cramping of the hands, and pain in the wrists occurred, and she was kept under the influence of quinine and anti-spasmodics. 19th, P. M., there was a return of the rigidity, with difficulty of respiration and deglutition. Treatment same, and she was soon relieved. During the night Doctor Dean, of Rochester, was called in consultation. He concurred in my views of the case and treatment. On the 20th she had her neuralgia at 11, A. M., but no cramps. She improved day by day, her neuralgia growing lighter and *later* each day. The tongue lost its dark congested hue. She suffered somewhat from muscular twitchings of the arms and from insomnia, but convalesced rapidly, a permanent contraction of the flexor muscles of the legs being the last symptom to disappear. This case, in many of its phenomena, resembles spinal meningitis. It was not in my judgment a case of hysteria.

Case 6. I shall not attempt to give from my note book a detailed history of this case, suffice it to say, that S. S. is a maiden lady, age unknown — has been for years under treatment by various physicians, for various ailments. For the last year she has been under my care. About a year since she fell in a fit, which I should suppose, from the description, to have been epileptic. Her spine was in its upper portion very tender. I counter-irritated, and attempted to regulate the digestive functions, which were much impaired. During the winter she had no return of the fit, and was most of the time under treatment for chronic gastritis, accompanied frequently by haematemesis. In the spring a slight prolapsus uteri was discovered, which was remedied by a pessary, and she was placed under successful treatment for menorrhagia. Her health apparently improved until August 5th, 1851, when she was found upon the floor insensible, and continued so for some two hours. On the 6th she was again found upon the floor, bloody froth oozing from her mouth. I saw her at 11, A. M., she had not spoken for two hours, was then insensible, eyes open, but she did not wink when they were touched; pupil answering to the light; jaws firmly set; her limbs powerless; no convulsion; head hot; countenance swollen and purple; pulse ninety. Cupped the nucha, and bled her, was able to get only ten ounces. Blistered neck and shoulders. At the end of an hour she answered questions by nodding, could not speak. Soon after spoke, complained of pain in the occiput; was numb; tongue stiff; swallowed with difficulty; tongue clean, but of a livid hue. Abdomen very tender; urine suppressed. She continued sensible until 7, P. M., when there was a return of insensibility with general and complete rigidity of the muscular system. These spasms have continued now for more than two months, in a form but little modified from that described above. I kept her for the first fortnight under a powerful revulsive and cathartic treatment, until one day she got up and dressed herself and went to work. She now, October 18th, has the "fits" every night and morning; her general health is good, and she assists considerably in household duties. I am now giving no medicines internally, and have a fine crop of tartar emetic pustules from her occiput to her sacrum. I intend to keep this up until I am satisfied it will do no good. It is difficult to tell upon what condition of the nervous system this depends. There was much heat about the head at first, but the general surface was cold. Afterwards there was considerable febrile action, which might have depended on the treatment. Her mind has not suffered, though she still complains of a dull pain in the occiput. She is clumsy in the use of her limbs, and says they are stiff and numb. Her spine is throughout very tender. Perhaps the gastric disturbance has something to do with the production of the spasms. Stokes, in his clinical lectures, gives cases of *convulsion* in adults, depending evidently on this cause. The uterine system was apparently in good order. During the spasm at times she rolled her head like a child in hydrocephalus, and her breathing was so catching and interrupted, as to suggest the idea of sub-arachnoid effusion.

Case 7. At 4, A. M., on the morning of the day on which I was called to the above case, I was called to Mr. R. H., a farmer, aged about forty-five years; found him dead. I gathered from the friends the following history of his case: He had been ill three or four weeks; was first attacked when in usual health, with insensibility. His physician supposed it to be a paralytic shock. The usual treatment was adopted with success, the patient becoming sensible, and it was then found that he had the control of his limbs. He had a strange feeling in his head, with neuralgic pain in the side of his head, occurring at intervals, and very severe. His general health seemed improving, until the night of his death the pain became severer. He bathed his feet, and after that, lying down on the bed he became insensible, his countenance pale and deathlike his breathing interrupted, long intervals occurring between respirations, and his pulse *continuing to beat for twenty minutes after the* last inspiration. The manner of death marks this as a case of compression of the base of the brain, and medulla oblongata. Probably the first shock was congestive, leaving behind it a little inflammatory action causing neuralgia — this inflammatory action resulting in sub-arachnoid effusion. The neuralgia under which Mr. H. suffered, was in the temporal branches of of the fifth pair. It is well to bear in mind that the fifth pair of nerves, although enumerated among the cranial nerves, are, in fact, spinal nerves in their functions.

Case 8, of which I have no notes, is somewhat similar in its sad result. A lady had suffered much from neuralgia, particularly of the fifth pair. While, as we had hoped, she was convalescing from a nursing sore mouth, accompanied by that crythematous inflammation of the mucous membranes of the primæ viæ, so difficult to manage, she was taken one evening with difficult inspiration. She acted a little strangely, but feeling better soon after, no attention was paid to the occurrence, which she attributed to wind on the stomach. The next morning, after a good night's rest, she got up, walking across the floor unassisted, and drank a glass of milk. While sitting in her chair the difficult respiration returned; she felt that she was dying; her countenance became anxious, (the pulse regular and full) and in two hours she expired. To what other cause was this sudden snapping of the silver cord to be attributed, than to sub-arachnoid effusion? And yet it was the first intimation we had of any cerebral disturbance, further than a slight tenderness of the cuvical vertebræ, which had existed for years.

Case 9. I was called, Aug. 18th, 1849, to a Miss I. S., for many years a confirmed epileptic, but still able to do some housework, and her mind untouched. For a few weeks back her fits had assumed a character somewhat resembling the descriptions of catalepsy. She felt an *aura* starting from the præcordia, and rising to the head; she became unable to speak, and after very slight convulsive movements, would assume a frightful staring look, while her limbs would remain for a few minutes in any position I placed them; then yielding to the laws of gravity, would fall upon the bed. She retained her consciousness during these spasms, which lasted from half an hour to an hour. Her spine in the whole cervical and dorsal region, was very tender. I regulated some little disorder of the digestive functions, and blistered her spine thoroughly, until Sept. 7th, when the epilepsy returned, and she was restored to her usual health. This occurred in Portage, Livingston Co. Leaving that section soon after, I lost track of the case, but have heard recently that the tonic spasm had returned, and she has become a hopeless idiot.

Case 10. The subject of this case was a young lady, Miss E. B., aged ty-five. She had been from childhood epileptic, the fits since puberty more frequent, occurring with each menstrual period. During the summer and fall they became of very frequent occurrence, complicated with the more than daily occurrence of spasm, or rather of momentary insensibility, some of these answering to the description of the *petit mal* of the French, others seemingly only continuations of the *petit mal*, but lasting for many minutes, during which she would perform the part of la somnambula, walking about and talking unconsciously, and on her return to reason very much surprised to find herself in a different locality from that in which the attack seized her. She became a little distrustful of her friends, and it was evident that a naturally good mind was breaking down under the assaults of disease. Her spine was somewhat tender in the cervical and interscapular regions. Her general health good. I prevailed on her, Oct. 21, 1850, to commence blistering the spine, as that afforded the only indication of treatment. Opiates and other narcotics, which she had used to quell neuralgic pain, were discontinued, and the blisters were continued till Dec. 23d, at intervals. The result was the cessation of both *grand* and *petit mal*, and she left here for Connecticut in the spring, having had, for three or four months, no return of her disease. I am informed that lately, owing to some fatigue and mental anxiety, the epileptic spasm has returned.

